# Dual Antiepileptics Induced Stevens-Johnson Syndrome: A Case Report

**DOI:** 10.31729/jnma.5308

**Published:** 2020-10-31

**Authors:** Prami Nakarmi, Sumit Raut, Siddhartha Manandhar, Abhash Shrestha

**Affiliations:** 1 Venus Hospital, Mid Baneshwor, Kathmandu, Nepal; 2 Kathmandu Medical College, Sinamangal, Kathmandu, Nepal; 3 Zheijang University School of Medicine, 866 Yu Hang Tang Lu, Xihu, Hangzhou, Zhejiang, P.R. China; 4 Chirayu National Hospital and Medical Institute, Basundhara, Kathmandu, Nepal

**Keywords:** *adverse drug reactions*, *antiepileptic drugs*, *case report*, *stevens-johnson syndrome*

## Abstract

Stevens-Johnson syndrome and Toxic Epidermal Necrolysis are acute mucocutaneous reactions hallmark of which is widespread necrosis and detachment of epidermis. SJS/TEN fall under the single disease spectrum with an incidence rate of 1.0 to 6.0 per 1000000 and 0.4 to 1.2 per 1000000 respectively. Here, we present a case of a 46 years female who developed a generalized erythematous rash over her body, 26 days after being exposed to phenytoin and sodium valproate. Given the strong association between SJS and antiepileptic drugs, and the usual presentation being within the first eight weeks of exposure to susceptible medications; we diagnosed her with SJS. Phenytoin and sodium valproate was withdrawn and she was managed with antihistamines and corticosteroids. She improved significantly within 15 days of our intervention. The mortality rates for SJS and TEN are up to 10% and 30-50% respectively. Early identification of SJS, discontinuation of triggering medicines, and prompt initiation of supportive therapy improve the prognosis.

## INTRODUCTION

Stevens-Johnson syndrome (SJS) is a type IV hypersensitivity reaction, involving the skin and mucous membrane, characterized by erythematous rash, erosions, and detachment of the epidermis, with an estimated incidence of 1.1 to 6.0 per million.^1^ It consists of macules, papules, or nodules which may later progress to purplish lesions, vesicles, bullae, or bleeding sites.^2^ Sodium valproate is a broad-spectrum antiepileptic drug used for a variety of psychiatric illnesses. Phenytoin, along with other aromatic antiepileptic drugs (AED) such as carbamazepine, phenobarbitone and primidone are known to cause hypersensitivity reactions usually within first 8 weeks of therapy.^3,4^ We present to you a case of Stevens-Johnson syndrome induced by sodium valproate and phenytoin.

## CASE REPORT

A 46-year-old woman initially presented in January 2020 with complaints of abnormal body movements, vomiting, and urine incontinence for one day. She also complained of feverish feeling with a maximum recorded temperature of 99 degree F and chills for the past few days. There was no history of trauma or previous similar episodes. However, she was a diagnosed case of hypertension and was taking Tab. Amlodipine 5mg for the last one year.

On arrival, her GCS was 9/15 and vital signs were within normal limits. After thorough clinical examination and investigations, a diagnosis of meningoencephalitis with pulmonary thromboembolism was made. Pulmonary thromboembolism was an incidental finding confirmed by a chest CT scan after a suspicious chest x-ray. The patient was managed in the intensive care unit and included intravenous fluids, antiemetics, anticoagulants, antibiotics, and antiepileptics (Inj. Phenytoin, Inj. Sodium valproate, Inj. Levetiracetam and Tab Clobazam). She was discharged after 17 days of hospital stay with anti-epileptics such as Tab. Sodium valproate, Tab. Phenytoin and Tab. Clobazam.

Her second visit to the hospital was after 11 days of her discharge, with the complaints of generalized body rash for the past three days. Initially erythematous maculopapular rash appeared on her trunk and later over her limbs, lips, face, associated with facial puffiness, difficulty in swallowing, and mild discomfort while breathing. However, she did not give any history of fever, cough, or previous similar episodes. Her family history was insignificant. Her vital signs were within normal limits.

On examination, erosions were present over the lips and oral mucosa, restricting mouth opening. Erythematous to dark-colored maculopapular rashes were present on her back, bilateral upper and lower limbs and face, involving less than 10% of total body surface area. There was no ocular involvement. Her blood parameters appeared normal. A diagnosis of Stevens-Johnson syndrome was made with a SCORTEN score of 2 (mortality rate of 12.1%). Phenytoin and sodium valproate were withdrawn from her prescription as the possible etiologies, but Clobazam was continued. The patient developed symptoms after 26 days of treatment with anti-epileptic drugs. Her symptoms subsided gradually with antihistamines and topical and systemic corticosteroids (Inj. Hydrocortisone 100mg IV TDS).

**Figure 1:**
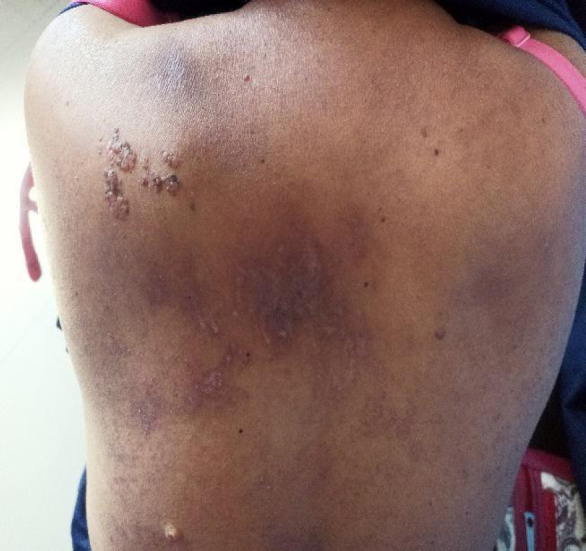
Fifteen days after initial appearance of rash over upper back.

**Figure 2:**
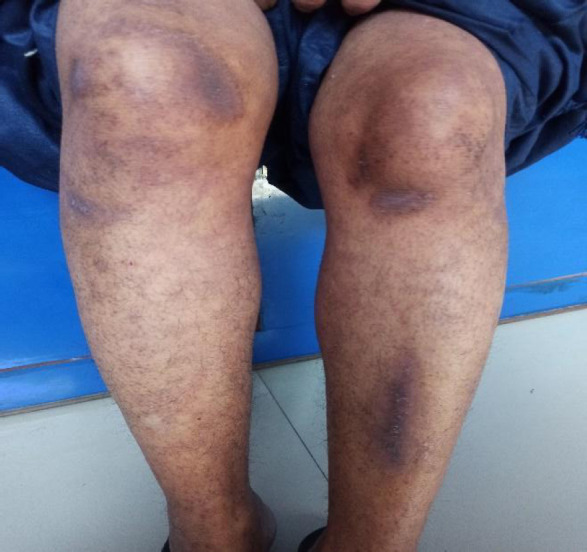
Fifteen days after initial appearance of rash over legs.

## DISCUSSION

Adverse drug reactions mostly present as cutaneous reactions manifesting as mild to severe life-threatening forms. Approximately 2-3% of hospitalized patients are found to have adverse drug reactions, out of which only 2% are of severe form.^5^ Severe cutaneous adverse reactions (SCAR) to drugs comprise of mainly Stevens-Johnson syndrome, Toxic Epidermal Necrolysis (TEN) and drug reaction with eosinophilia and systemic symptoms (DRESS) syndrome.^6^

About 30-50% cutaneous drug reaction cases are Stevens-Johnson syndrome (SJS), and 80% are Toxic Epidermal Necrolysis (TEN).^7^ SJS and TEN involve the skin and the mucous membrane, differentiated by the extent of epidermal detachments (<10% total body surface area in SJS, 10-30% total body surface area involvement in SJS/TEN overlap syndrome, while, more extensive, >30% of total body surface area in TEN) and higher mortality in TEN (30-50%) as compared to SJS (1-9%).^6^ Still, the mortality of 16.39% for SJS has also been reported in an Indian population.^7,8,9,10^ SCORTEN scale is a predictor of the mortality rate in SJS/TEN patients and is calculated by factors such as age of the patient, tachycardia, total body surface area involved, increased serum urea, increased serum glucose and increase bicarbonate levels.^11^ A score of 5 or more indicates a mortality rate of 90%.^11^

SJS frequently occurs within the first eight weeks of therapy as a response to certain drugs, commonly the antiepileptics (carbamazepine, phenobarbital, phenytoin, lamotrigine, sodium valproate), antibiotics (sulfonamides, penicillin, macrolides, fluoroquinolone, tetracycline), antitubercular drugs (ethambutol, rifampicin), NSAIDs.^4,10^ Sodium valproate monotherapy induced SJS is rare, but cases have been reported. Valproate is more likely to cause SJS when given in combination with other antiepileptic drugs.^4^ In our case report, the patient was prescribed phenytoin as well as valproate, and the symptoms of cutaneous drug reaction started after 26 days following the treatment.

In a literature review by Letko E et al., the collected information estimated the incidence of SJS as 1.1-7.1/1000000 people per year.^12^ A case-control study done in Taiwan revealed an incidence rate of SJS, ranging between 3.3-4.1/1000000 people per year.^4^ The same study evaluated 7327 patients with an established diagnosis of SJS; it revealed an increased association with the use of drugs: 21.3% (1560) for allopurinol, 16.7% (1223) for carbamazepine and 9.1% (667) for phenytoin, in comparison to a control group.^4^ Among the 667 patients who took phenytoin, 83 (12.4%) cases had also taken valproate.^4^

Aromatic amine antiepileptics (carbamazepine, phenytoin, and phenobarbital) are metabolized to arene oxide metabolites resulting in cellular toxicity.^13^ The presence of specific HLA alleles such as HLA-B*15:02, HLA-B*15:11, HLA-B*59:01 has also been reported to increase susceptibility to adverse drug reactions due to antiepileptic drugs.^11,14^

SJS can present with fever, malaise, sore throat, cough, mucocutaneous lesions with blisters, and other systemic symptoms such as eye redness which may even result in visual impairment.^15^ The initial non-specific symptoms may mislead the diagnosis, hence delaying the treatment. The primary treatment comprises of immediate identification and withdrawal of the offending drug followed by medication and supportive care.^14^ Medical treatment include corticosteroids, intravenous immunoglobulin (IVIG), cyclosporine, TNF inhibitors combined with proper wound care.^14^

The use of corticosteroids is controversial as some studies suggest using it in the early phases while others are against the use as it is associated with adverse effects and increased duration of recovery.^16,17,18^ The study by Rasmussen shows increased hospital stay and side effects in patients who received corticosteroids in their treatment in comparison to patients who were not given corticosteroids.^16^ Kardaun and Jonkman states that high doses of corticosteroids given for a short duration in early SJS/TEN is beneficial and the adverse effects seen with corticosteroids use is probably due to late administration, low dose and prolonged use.^18^

SJS can result in a fatal, life-threatening condition if not recognized early; therefore, importance should be driven when susceptible drugs are prescribed either alone or in combination.

## Consent:

**JNMA Case Report Consent Form** was signed by the patient and the original article is attached with the patient's chart.

## Conflict of Interest

**None.**
